# A Decade of Durable Mechanical Circulatory Support with HeartMate 3 Across Age Groups in a Single-Center Experience

**DOI:** 10.3390/life16101640

**Published:** 2026-09-30

**Authors:** Sedat Karaca, Umit Kahraman, Aysen Yaprak Kapkin, Ahmet Daylan, Osman Nuri Tuncer, Irem Demiray, Cagatay Engin, Tahir Yagdi

**Affiliations:** 1 Department of Cardiovascular Surgery, Sanliurfa Training and Research Hospital, Sanliurfa 63250, Türkiye; karacasedat34@gmail.com; 2 Department of Cardiovascular Surgery, Ege University Medical School, Izmir 35100, Türkiye; umit.kahraman@ege.edu.tr (U.K.); aysen.yaprak.kapkin@ege.edu.tr (A.Y.K.); ahmet.daylan@ege.edu.tr (A.D.); osman.nuri.tuncer@ege.edu.tr (O.N.T.); irem.demiray@ege.edu.tr (I.D.); cagatay.engin@ege.edu.tr (C.E.)

**Keywords:** heart failure, left ventricular assist device, HeartMate 3, LVAD

## Abstract

Background: Long-term real-world data after HeartMate 3 implantation remain limited, particularly regarding age-specific outcomes. We evaluated on-device survival and major adverse events across nearly a decade of experience. Methods: This retrospective single-center study included 215 patients undergoing primary HeartMate 3 implantation between January 2016 and October 2025. On-device mortality was analyzed using Aalen–Johansen cumulative incidence estimates with heart transplantation as a competing event; age-group differences were assessed using Fine–Gray regression. Transplant-censored Kaplan–Meier survival was examined secondarily, and mortality predictors were evaluated using multivariable Cox regression. Results: Median age was 53 years and median support was 603 days. At data cutoff, 149 patients remained alive on support, eight underwent transplantation, and 58 died on support. At 5 years, the cumulative incidences of on-device death and heart transplantation were 35.3% and 4.1%, respectively, with no significant age-group difference (Fine–Gray global *p* = 0.088). In the secondary transplant-censored Kaplan–Meier analysis, estimated survival was 87.4% at 30 days, 81.1% at 1 year, and 63.8% at 5 years. Higher preoperative creatinine was associated with mortality (HR, 1.93 per 1-mg/dL increase; 95% CI, 1.07–3.50; *p* = 0.029). No intrinsic pump thrombosis occurred. Conclusions: HeartMate 3 provided durable support with favorable long-term survival, although non-pump adverse events remained clinically relevant.

## 1. Introduction

Durable left ventricular assist devices (LVADs) have become an established therapeutic option for selected patients with advanced heart failure, either as a bridge to transplantation or as long-term support. Contemporary guidelines recommend durable mechanical circulatory support in appropriately selected patients with advanced heart failure when conventional medical therapy is insufficient [1,2]. Over the past two decades, technological advances in continuous-flow LVADs have progressively improved survival and reduced device-related morbidity.

The HeartMate 3 (Abbott, Chicago, IL, USA) is a centrifugal-flow LVAD incorporating a fully magnetically levitated rotor, wide blood-flow pathways, and programmed speed modulation designed to improve hemocompatibility and pump durability. In the MOMENTUM 3 trial, HeartMate 3 was associated with superior long-term outcomes compared with the axial-flow HeartMate II, including higher 5-year survival and substantially lower rates of pump thrombosis, stroke, and bleeding [3]. Contemporary registry data also demonstrate the near-complete transition toward fully magnetically levitated durable LVAD technology, with 99.8% of primary LVAD implants recorded in the 2022 STS-INTERMACS cohort using this platform [4].

Despite these advances, evidence describing very long-term HeartMate 3 support remains comparatively limited. MOMENTUM 3 established randomized comparative efficacy and provided extended 5-year follow-up, while large registries such as STS-INTERMACS and prospective real-world studies including ELEVATE have characterized outcomes in broader populations [3,4,5]. However, single-center real-world cohorts extending beyond 5 years remain uncommon, particularly those describing prolonged on-device survival together with major complications over nearly a decade of clinical practice. Such longitudinal experience may complement randomized and multicenter registry data by characterizing outcomes during sustained device support in routine clinical care.

Age is also clinically relevant in contemporary LVAD practice because increasing use of long-term and destination support has expanded treatment to older patients with a greater burden of comorbidity and potentially different competing risks and adverse-event profiles [6]. Evaluating outcomes across age groups may therefore help distinguish the influence of chronological age from other preimplant clinical characteristics when considering durable mechanical circulatory support.

Accordingly, we hypothesized that HeartMate 3 would provide durable long-term support across age groups, while patient-related factors rather than chronological age alone would account for differences in on-device mortality. We therefore evaluated transplant-censored on-device survival, competing outcomes, major adverse events, and predictors of mortality in patients undergoing primary HeartMate 3 implantation at our institution over a nearly decade-long period.

## 2. Materials and Methods

### 2.1. Study Design and Patient Population

This retrospective observational cohort study included all consecutive patients who underwent primary HeartMate 3 (Abbott, Chicago, IL, USA) implantation at a high-volume tertiary heart failure surgery center between January 2016 and October 2025. During the study period, a total of 236 patients underwent HeartMate 3 implantation. Of these, 12 patients who received HeartMate 3 as part of biventricular assist device (BiVAD) support and 9 patients who underwent HeartMate 3 implantation as a pump exchange procedure were excluded, resulting in a final study cohort of 215 patients. Clinical follow-up was updated through 1 June 2026. Final device status was available for all 215 patients, who were classified at the end of observation as alive on ongoing HeartMate 3 support, having undergone heart transplantation, or having died during device support. A STROBE-style study flow diagram summarizing patient selection and exclusion is provided in Supplementary Figure S1.

Treatment strategy was determined by the multidisciplinary advanced heart failure team according to transplant eligibility, age, comorbidities, end-organ function, and overall clinical status. Patients aged ≤ 70 years were generally evaluated for heart transplantation and, when otherwise eligible, HeartMate 3 implantation was considered primarily as a bridge-to-transplant strategy. In patients aged > 70 years or in those considered unsuitable for transplantation because of relevant comorbidities or other clinical factors, durable LVAD support was generally used as destination therapy. Chronological age alone was not considered an absolute determinant of treatment strategy, and individual clinical assessment remained decisive.

The study was conducted in accordance with the STROBE recommendations. The study protocol was approved by the institutional ethics committee (approval no: 25-12T/15), and written informed consent for the use of anonymized clinical data for research purposes was obtained from all patients.

### 2.2. Data Collection and Follow-Up

Baseline demographic, clinical, operative and postoperative data were collected from institutional electronic medical records and the institutional mechanical circulatory support database. Long-term follow-up data, including device status, hospital readmissions, and major device-related adverse events, were obtained from scheduled LVAD outpatient visits and institutional hospital records.

Following hospital discharge, patients were routinely evaluated at 1 week, 2 weeks, and 1 month, and subsequently at 3-month intervals in the dedicated LVAD outpatient clinic. Additional evaluations were performed whenever clinically indicated. No patient was lost to follow-up, and final device status was available for the entire study cohort.

Patients were followed from the date of HeartMate 3 implantation until death on LVAD support, heart transplantation or the last available device-related clinical follow-up. The duration of LVAD support was calculated from the date of implantation to death, heart transplantation or last follow-up, as appropriate.

### 2.3. Surgical Procedure

All HeartMate 3 implantations were performed using a standardized surgical technique at our institution. All patients underwent device implantation via median sternotomy under cardiopulmonary bypass. In patients undergoing redo sternotomy, peripheral cannulation was used when deemed necessary according to the surgical team’s judgement.

The HeartMate 3 pump was implanted at the left ventricular apex using the standard sewing ring technique. The outflow graft was anastomosed end-to-side to the ascending aorta. Concomitant surgical or interventional procedures were performed when clinically indicated.

Pump speed was individualized using intraoperative and postoperative hemodynamic and echocardiographic assessment, with the goals of achieving adequate left ventricular unloading and systemic perfusion while maintaining the interventricular septum in a midline position, minimizing mitral regurgitation, and allowing intermittent aortic valve opening when clinically appropriate. Pump speed was subsequently adjusted during follow-up according to clinical and echocardiographic findings.

### 2.4. Antithrombotic Management

Throughout the study period, vitamin K antagonist therapy was used with a target international normalized ratio (INR) of 2.0–3.0. Enoxaparin bridging was continued until the therapeutic INR range was achieved. Aspirin 100 mg daily was routinely used in combination with vitamin K antagonist therapy during the earlier study period, whereas vitamin K antagonist monotherapy without routine aspirin was increasingly adopted in later years in accordance with evolving evidence and clinical practice. Antithrombotic therapy was individualized when clinically indicated, particularly in the setting of bleeding or thromboembolic events.

### 2.5. Study Outcomes

The primary outcome of the study was on-device all-cause mortality during HeartMate 3 support. In the primary time-to-event analysis, heart transplantation was treated as a competing terminal event.

Secondary outcomes included heart transplantation, transplant-censored on-device survival, and major device-related adverse events during follow-up, including major neurological events, gastrointestinal bleeding, driveline infection, device failure, and clinically significant arrhythmia during follow-up. Early postoperative outcomes, including early postoperative right heart failure and early postoperative arrhythmia, were evaluated separately.

Primary and secondary outcomes were evaluated in the overall cohort and according to the prespecified age groups at implantation (<50 years, 50–64 years, and ≥65 years).

### 2.6. Definition of Parameters

Adverse-event definitions were based, where applicable, on contemporary mechanical circulatory support consensus definitions [7].

*Major neurological event*: Defined as the occurrence of ischemic stroke or intracranial hemorrhage during follow-up.

*Gastrointestinal bleeding*: Defined as clinically significant bleeding originating from the gastrointestinal tract requiring hospitalization, blood transfusion, endoscopic or interventional treatment, or resulting in hemodynamic compromise.

*Driveline infection*: Defined according to contemporary ISHLT criteria as an uncomplicated or complicated percutaneous lead infection involving the driveline exit site [8]. Clinical features included pain, tenderness, erythema, drainage, and/or induration at the driveline site. Drainage, tissue, and blood cultures were obtained when clinically indicated; a positive local culture supported the diagnosis but was not mandatory for uncomplicated infection. Evidence of abscess or deeper extension on imaging, systemic manifestations, bloodstream infection, or positive deep tissue cultures was considered indicative of complicated infection.

*Early postoperative right heart failure*: Defined as new or persistent right ventricular dysfunction following HeartMate 3 implantation, documented by the advanced heart failure/cardiology team on the basis of clinical and echocardiographic assessment, and resulting in the need for temporary right-sided mechanical circulatory support, extracorporeal membrane oxygenation for right-sided circulatory failure, or prolonged inotropic or inhaled pulmonary vasodilator support extending beyond 14 days after implantation.

*Device failure*: Defined as device-related mechanical complication requiring pump exchange, urgent device replacement, or intervention for clinically significant outflow graft obstruction.

*Pump thrombosis:* Defined as suspected or confirmed thrombus involving the device blood-flow pathway, including the inflow cannula, pump rotor, or outflow graft, in accordance with contemporary guideline criteria [7]. Diagnosis was based on the integrated assessment of device parameter abnormalities, clinical evidence of impaired device function, laboratory evidence of hemolysis, echocardiographic findings including inadequate left ventricular unloading, and imaging or operative confirmation when available.

*Early postoperative arrhythmia*: Defined as a clinically significant atrial or ventricular arrhythmia occurring during the index postoperative hospitalization and requiring specific rhythm-directed treatment, including electrical cardioversion or catheter ablation.

*Arrhythmia during follow-up*: Defined as a clinically significant arrhythmia documented during subsequent HeartMate 3 support after the early postoperative period. Because detailed arrhythmia subtype, recurrent arrhythmia burden, ICD therapies, and associated hemodynamic consequences were not systematically available throughout the study period, this outcome was analyzed as patient-level occurrence rather than according to arrhythmia subtype or recurrent-event burden.

### 2.7. Statistical Analysis

Continuous variables are summarized as median with interquartile range (IQR), and categorical variables as counts and percentages. For the prespecified exploratory age-stratified analysis, patients were categorized as <50, 50–64, and ≥65 years according to age at implantation. These age categories were prespecified and selected in accordance with previously published LVAD registry analyses using the same stratification, with the aim of distinguishing younger, middle-aged, and older recipients for exploratory outcome comparisons rather than defining formal bridge-to-transplantation or destination-therapy thresholds [6]. Continuous variables were compared across age groups using the Kruskal–Wallis test. Categorical variables were compared using the Pearson chi-square test when expected cell counts were adequate and the Fisher–Freeman–Halton exact test when the assumptions of the chi-square test were not met. All age-group comparisons were considered exploratory, and no adjustment for multiple comparisons was performed.

No formal a priori sample-size calculation was performed, because this retrospective cohort comprised all consecutive eligible patients who underwent primary HeartMate 3 implantation during the prespecified study period.

On-device mortality was analyzed within a competing-risk framework. Death occurring during HeartMate 3 support and heart transplantation were considered mutually exclusive terminal events. For the cumulative incidence of on-device death, heart transplantation was treated as a competing event; conversely, on-device death was treated as a competing event when estimating the cumulative incidence of transplantation. Cumulative incidence functions were estimated using the Aalen–Johansen method. Patients who remained alive on HeartMate 3 support were administratively censored at the date of last follow-up or the study cutoff date. Differences in the cumulative incidence of on-device death among age groups were evaluated using Fine–Gray subdistribution hazards regression.

Transplant-censored on-device survival was also estimated using the Kaplan–Meier method as a secondary descriptive analysis to provide conventional time-specific survival benchmarks and facilitate contextual comparison with previous HeartMate 3 studies. Patients undergoing heart transplantation were censored at the transplantation date.

Cause-specific multivariable Cox proportional hazards regression was used to evaluate factors associated with on-device mortality. Covariates were selected a priori on the basis of clinical relevance and included age per 10-year increase, sex, ischemic etiology, INTERMACS profile 1–2, preoperative creatinine, and preoperative hemoglobin, inotropic support, and implantation year. All covariates were entered into the model simultaneously, without data-driven variable selection. Hazard ratios (HRs) with 95% confidence intervals (CIs) were reported. The linearity assumption for continuous covariates was assessed using restricted cubic spline terms, with no evidence of a nonlinear association for preoperative creatinine.

The proportional hazards assumption was assessed using Schoenfeld residuals, and the linearity of continuous covariates was evaluated before model interpretation. There were no missing data for the covariates included in the final multivariable model; therefore, no imputation was required. Conventional statistical analyses were performed using SPSS version 25.0 (IBM Corp., Armonk, NY, USA). Competing-risk analyses and independent verification of the survival models were performed using Python version 3.14.6 (Python Software Foundation, Wilmington, DE, USA). statistical routines. All tests were two-sided, and *p* < 0.05 was considered statistically significant.

## 3. Results

### 3.1. Patient Population and Device-Related Outcomes

A total of 215 consecutive patients who underwent primary HeartMate 3 LVAD implantation between January 2016 and October 2025 were included in the final analysis. The data cutoff date for survival follow-up was 1 June 2026. At the last device-related follow-up, 149 patients (69.3%) were alive on ongoing LVAD support, eight patients (3.7%) had undergone heart transplantation, and 58 patients (27.0%) had died while receiving LVAD support. The median observed duration of LVAD support was 603 days (IQR, 188–1210) (range, 2–3617 days), corresponding to 1.65 years (IQR, 0.51–3.31). The median follow-up estimated using the reverse Kaplan–Meier method was 1061 days (2.91 years; IQR, 559–2044 days).

### 3.2. Baseline Characteristics

Baseline characteristics according to age group are summarized in Table 1. Of the 215 patients, 85 (39.5%) were younger than 50 years, 109 (50.7%) were 50–64 years, and 21 (9.8%) were aged 65 years or older. The distribution of heart failure etiology differed significantly across age groups (*p* = 0.003), with ischemic cardiomyopathy becoming more frequent with increasing age (20.0%, 44.0%, and 57.1%, respectively). Baseline renal function was also less favorable in the older age groups, with higher creatinine (*p* = 0.001) and lower eGFR (*p* < 0.001), while hemoglobin levels differed significantly across groups (*p* = 0.003). INTERMACS profile distribution did not differ significantly according to age (*p* = 0.126). VAD treatment strategy differed markedly according to age (*p* < 0.001), with destination therapy accounting for 57.1% of implants in patients aged ≥ 65 years compared with 7.3% in those aged 50–64 years and none in patients aged < 50 years. Systolic pulmonary artery pressure also differed modestly across age groups (*p* = 0.043), although there was no consistent monotonic increase with advancing age. No post-hoc pairwise comparisons were performed.

### 3.3. Operative and Early Postoperative Outcomes

Operative and early postoperative outcomes according to age group are presented in Supplementary Table S1. The median cardiopulmonary bypass time was 69 min (IQR, 57–84), and redo sternotomy was performed in 28 patients (13.0%). The median ICU and hospital stays were 9 days (IQR, 7–13) and 23 days (IQR, 19–30), respectively. Concomitant procedures included aortic valve replacement in 16 patients, coronary artery bypass grafting in 2, tricuspid ring annuloplasty in 1, and patent foramen ovale closure in 4. Thirty-day on-device mortality was 12.6% (27/215). Early postoperative right heart failure differed significantly across age groups, occurring in 20.0% of patients aged < 50 years, 25.7% of those aged 50–64 years, and 47.6% of those aged ≥ 65 years (*p* = 0.036). No other major early postoperative outcome showed a statistically significant difference across age groups. Global comparisons across age categories are reported as exploratory analyses.

### 3.4. Follow-Up Findings and Device-Related Adverse Events

Follow-up status and major device-related adverse events according to age group are summarized in Table 2. At the data cutoff, 149 patients (69.3%) remained alive on ongoing LVAD support, eight (3.7%) had undergone heart transplantation, and 58 (27.0%) had died while receiving LVAD support. No intrinsic pump thrombosis occurred. Seven patients experienced device failure, including outflow graft-related obstruction or other mechanical problems. Major neurological events affected 14 patients and comprised 8 ischemic and 6 hemorrhagic stroke events. Driveline infection occurred in 45 patients (20.9%), and arrhythmia during follow-up was documented in 23 patients (10.7%). Gastrointestinal bleeding differed significantly across age groups, occurring in 5.9%, 7.3%, and 23.8% of patients aged < 50, 50–64, and ≥65 years, respectively (global *p* = 0.025). No post-hoc pairwise comparisons were performed.

### 3.5. Survival Analysis

**Figure 1:**
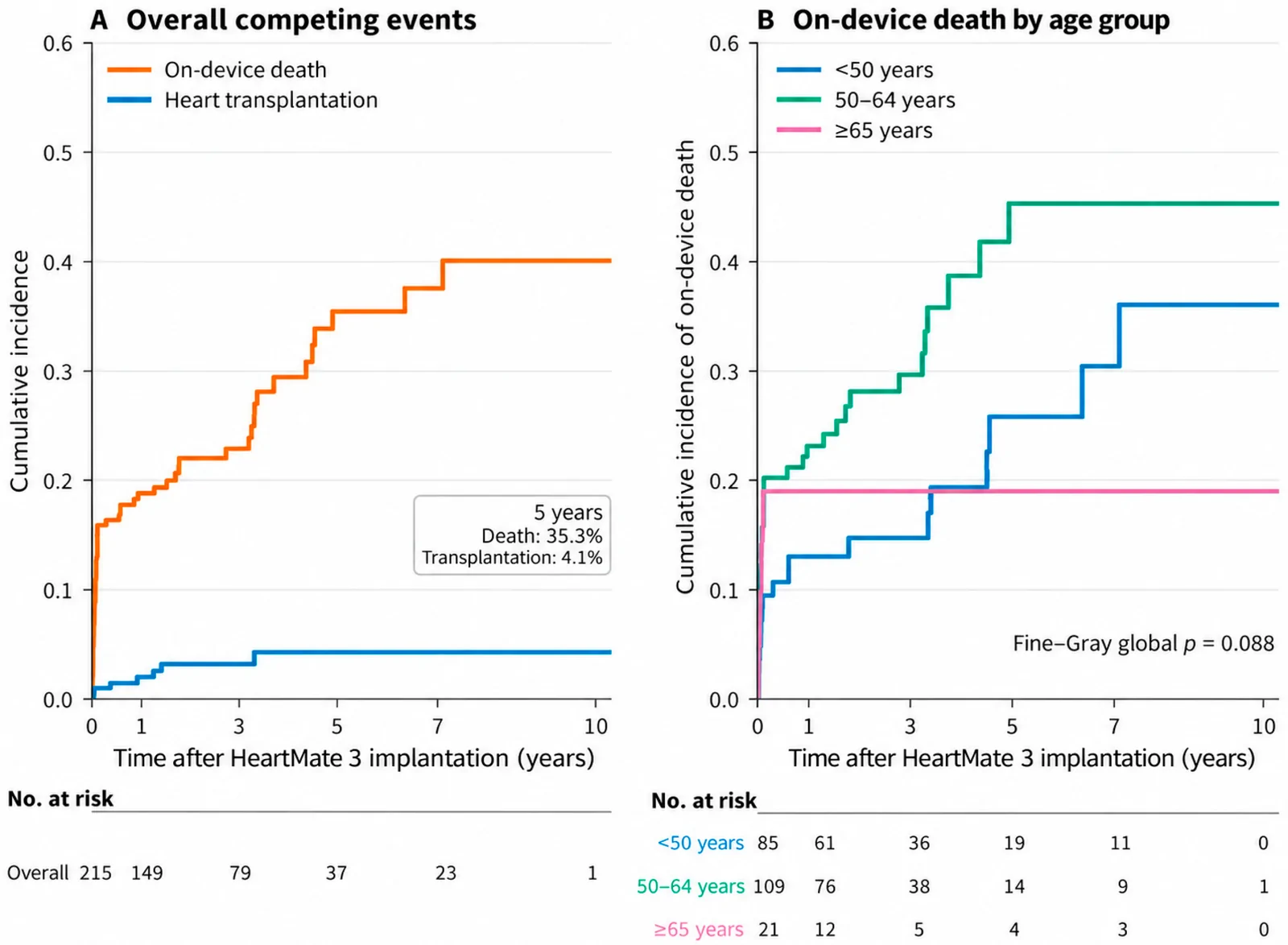
Cumulative incidence of on-device death and heart transplantation in the overall cohort (**A**) and on-device death according to age group (**B**).

In the competing-risk analysis, the 5-year cumulative incidence of on-device death was 35.3%, whereas the corresponding cumulative incidence of heart transplantation was 4.1%. The estimated probability of remaining alive on HeartMate 3 support without transplantation at 5 years was therefore 60.5%. The cumulative incidence of on-device death did not differ significantly among the three age groups (Fine–Gray global *p* = 0.088) (Figure 1). Although the estimate in patients aged ≥ 65 years appeared numerically favorable, this subgroup included only 21 patients, with four patients remaining at risk at 5 years; therefore, no firm conclusions regarding the effect of age can be drawn from this exploratory comparison.

**Figure 2:**
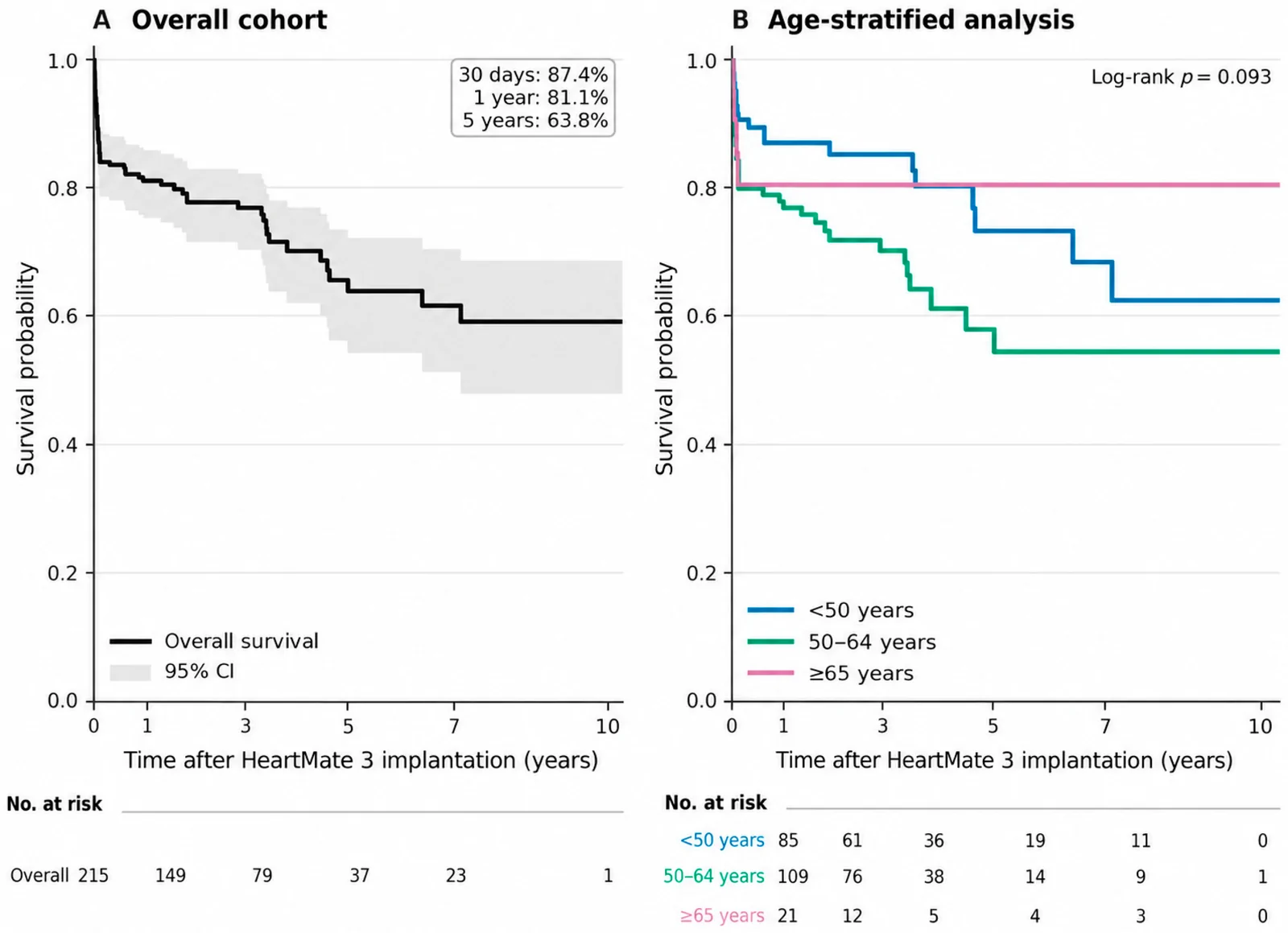
Transplant-censored Kaplan–Meier survival in the overall cohort (**A**) and according to age group (**B**).

In the secondary transplantation-censored Kaplan–Meier analysis, estimated on-device survival was 87.4% (95% CI, 82.1–91.2) at 30 days, 81.1% (95% CI, 75.2–85.8) at 1 year, and 63.8% (95% CI, 53.9–72.1) at 5 years (Figure 2).

### 3.6. Multivariable Analysis of On-Device Mortality

The multivariable Cox proportional hazards model included all 215 patients and 58 deaths occurring during LVAD support. Age was not statistically associated with transplant-censored on-device mortality when evaluated per 10-year increase (HR 1.15, 95% CI 0.87–1.52; *p* = 0.338). Higher preoperative creatinine was independently associated with mortality (HR 1.93 per 1 mg/dL increase, 95% CI 1.07–3.50; *p* = 0.029). There was no evidence of nonlinearity in the association between preoperative creatinine and on-device mortality when assessed using restricted cubic splines (*p* for nonlinearity > 0.90). Lower preoperative hemoglobin showed a borderline association with mortality but did not reach statistical significance (HR 0.88 per 1 g/dL increase, 95% CI 0.76–1.01; *p* = 0.076). Sex, ischemic cardiomyopathy, and INTERMACS profile 1–2, inotropic support, and implantation year were not statistically associated with on-device mortality (Table 3). The proportional-hazards assumption was assessed using Schoenfeld residuals. No evidence of violation was identified for individual covariates or for the model globally (global Schoenfeld test, *p* = 0.862; all covariate-specific *p* > 0.20). The multivariable Cox model demonstrated modest discrimination, with a Harrell C-statistic of 0.648.

## 4. Discussion

The present study summarizes nearly a decade of single-center experience with the HeartMate 3 left ventricular assist device and provides real-world evidence regarding its long-term durability and clinical outcomes. The principal findings were encouraging long-term survival, the complete absence of pump thrombosis, and sustained device support extending over several years in a substantial proportion of patients. Nevertheless, clinically relevant adverse events, particularly neurological complications, bleeding, driveline infection, and device failure, continued to accumulate during prolonged support. These findings demonstrate both the substantial progress achieved with the HeartMate 3 platform and the complications that remain relevant as patients live longer with durable mechanical circulatory support.

Long-term survival in the present cohort was consistent with the favorable outcomes reported for contemporary fully magnetically levitated centrifugal-flow devices. Transplant-censored on-device survival was 81.1% at 1 year and 63.8% at 5 years. In the randomized MOMENTUM 3 trial, Mehra et al. reported an overall 5-year survival of 58.4% among patients supported with HeartMate 3, while the prospective real-world ELEVATE registry demonstrated a 5-year survival of 63.3% in its primary implant cohort [3,5]. Although direct comparisons should be interpreted cautiously because of differences in patient selection, transplantation rates, follow-up completeness, endpoint definitions, and censoring strategies, the broadly consistent long-term estimates provide additional real-world support for the durability of HeartMate 3 therapy outside controlled clinical trials. However, these long-term estimates should be interpreted in the context of substantial censoring, as 149 of 215 patients (69.3%) remained alive on ongoing HeartMate 3 support at the data cutoff. Although the reverse Kaplan–Meier median follow-up was 2.91 years, the number of patients remaining under observation decreased progressively at later time points and estimates toward the end of follow-up are therefore less precise. These findings may partly reflect the device’s fully magnetically levitated rotor, wide blood-flow pathways, reduced shear stress, and programmed speed modulation intended to generate an artificial pulse, which collectively improve hemocompatibility and long-term pump durability.

In the exploratory age-stratified analysis, patients younger than 50 years demonstrated favorable on-device survival, with estimated survival rates of 86.9% at 1 year and 73.3% at 5 years. These findings are consistent with contemporary evidence supporting favorable outcomes with HeartMate 3 support in younger adults. In a propensity-matched analysis of patients aged 18–49 years, Uriel et al. reported comparable 2-year survival after HeartMate 3 implantation and heart transplantation (88.7% vs. 90.2%) [9]. The age-stratified findings warrant cautious interpretation. Patients aged ≥ 65 years represented only 21 patients in the present cohort and differed substantially from younger recipients in treatment strategy, ischemic etiology, renal function, and hemoglobin level. Moreover, only four patients in this subgroup remained at risk at 5 years. Although the cumulative incidence of on-device death appeared numerically favorable in the oldest group, this estimate is therefore imprecise and should not be interpreted as evidence of superior outcomes or of the absence of an age-related effect. A separate contemporary analysis focusing on middle-aged and older HeartMate 3 recipients likewise reported lower 2-year survival among patients aged ≥ 65 years than among those aged 50–64 years [10]. Differences in age thresholds, patient selection, treatment strategy, transplantation, and endpoint definitions preclude direct comparison with the present cohort. Nevertheless, these larger studies emphasize that the absence of a statistically significant association in our relatively small cohort should not be interpreted as evidence that chronological age has no prognostic relevance. Rather, our age-stratified findings should be regarded as exploratory and interpreted in conjunction with the broader clinical profile and competing treatment pathways of individual patients. Treatment intent was particularly age-dependent: destination therapy accounted for 57.1% of implants in patients aged ≥ 65 years, compared with 7.3% in those aged 50–64 years and none in patients aged < 50 years. A similar pattern has been reported in the PCHF-VAD Registry, in which destination therapy accounted for 61.1% of implants among patients aged ≥ 65 years [6]. This distribution reflects the close relationship between chronological age, transplant eligibility, and LVAD strategy and further limits interpretation of age-stratified outcomes as an isolated effect of age. Dilated cardiomyopathy accounted for 62.8% of the overall cohort, while ischemic cardiomyopathy became progressively more frequent across the age groups, indicating that the observed age comparisons also reflect differences in underlying heart failure etiology and institutional case mix. Systolic pulmonary artery pressure differed modestly across age groups; however, the absence of a consistent monotonic pattern with increasing age suggests that this finding should be interpreted cautiously rather than as a specific age-related hemodynamic effect.

Renal function emerged as an important preoperative factor associated with mortality in our cohort. Each 1 mg/dL increase in preoperative creatinine was associated with a 93% higher hazard of transplant-censored on-device death (HR 1.93, 95% CI 1.07–3.50; *p* = 0.029). This finding is consistent with previous LVAD studies demonstrating poorer long-term outcomes in patients with impaired preimplant renal function and with contemporary HeartMate 3 data identifying renal indices as relevant predictors of 5-year mortality [11,12]. Preoperative renal dysfunction may reflect both the severity of cardiorenal syndrome and reduced physiological reserve, potentially increasing susceptibility to perioperative kidney injury and multiorgan dysfunction. Higher preoperative hemoglobin was directionally associated with lower mortality (HR 0.88 per 1 g/dL increase, 95% CI 0.76–1.01), but this association did not reach statistical significance (*p* = 0.076) and should therefore not be interpreted as an independent protective effect. These findings support careful renal and hematologic assessment during LVAD evaluation, although the observational design precludes causal conclusions regarding the benefit of preoperative correction.

A particularly important finding was the absence of pump thrombosis throughout the entire follow-up period. Pump thrombosis was one of the most consequential complications of earlier-generation continuous-flow devices, potentially leading to hemolysis, systemic thromboembolism, pump exchange, or death. In the 5-year analysis of the MOMENTUM 3 trial, pump thrombosis occurred in only 2.1% of HeartMate 3 recipients, corresponding to 0.01 events per patient-year, compared with 17.6% and 0.108 events per patient-year among patients supported with the axial-flow HeartMate II device [3]. Similarly, the ELEVATE registry reported suspected pump thrombosis in only 1.1% of patients (n = 5) during 5 years of real-world follow-up, whereas no pump thrombosis was observed in the initial 5-year HeartMate 3 CE Mark experience [5,13]. Its complete absence in our cohort is especially notable given the prolonged duration of support in many patients, during which cumulative exposure to device-related thrombotic complications would normally be expected to increase. This result provides further real-world evidence of the favorable hemocompatibility and thrombotic safety profile of the HeartMate 3 and supports the view that intrinsic pump thrombosis is now an uncommon complication of contemporary HeartMate 3 support.

However, the absence of pump thrombosis does not imply freedom from mechanical device-related complications. In the present study, outflow graft obstruction and other mechanical problems were classified under the broader category of device failure rather than pump thrombosis. This distinction is important because outflow graft obstruction may result from graft twisting, kinking, anastomotic narrowing, intraluminal thrombus, or external compression caused by the progressive accumulation of acellular biodebris between the outflow graft and its bend relief and therefore does not necessarily represent thrombosis within the pump itself. In an international analysis involving 2108 HeartMate 3 recipients from 17 centers across eight countries, Wert et al. identified external outflow graft obstruction in 62 patients, corresponding to an overall prevalence of 3.0%. The median duration of support at diagnosis was 953 days, and the estimated incidence increased from 0.6% at 1 year to 9.1% at 5 years, highlighting its predominantly late presentation [14]. Similarly, Milwidsky et al. reported outflow graft obstruction in 5 of 83 HeartMate 3 recipients, with an institutional incidence of 7% and an event rate of 0.05 per patient-year; all events involved the bend relief-covered segment, and external compression appeared to be the predominant mechanism [15]. Ajello et al. subsequently described extrinsic obstruction in 5 of 41 HeartMate 3 recipients after 607–1250 days of support, without evidence of endoluminal thrombosis, further illustrating the insidious and predominantly late nature of this complication [16]. Therefore, although the HeartMate 3 has substantially reduced intrinsic pump-related thrombotic events, continued surveillance for unexplained low-flow alarms, declining estimated flow, inadequate ventricular unloading, recurrent heart failure symptoms, and outflow graft abnormalities remains essential during long-term follow-up. Computed tomography angiography, supplemented by conventional angiography or intravascular ultrasound, when necessary, should be considered when outflow graft obstruction is clinically suspected.

Neurological complications remained a major source of morbidity in our cohort. Major neurological complications affected 14 patients, comprising 8 ischemic and 6 hemorrhagic stroke events. The persistence of cerebrovascular events despite the complete absence of pump thrombosis emphasizes that improved pump hemocompatibility does not eliminate neurological risk during long-term LVAD support. In the prospective multicenter HeartMate 3 CE Mark trial, stroke occurred in 18% of patients during the first year, with an almost equal distribution between ischemic and hemorrhagic events (10% and 8%, respectively), despite the absence of pump thrombosis or clinically significant hemolysis [17]. Several of these events were associated with invasive procedures or infection, while one fatal intracerebral hemorrhage occurred in the setting of an international normalized ratio greater than 6, illustrating the heterogeneous mechanisms underlying neurological complications [17]. Similarly, in the seven-center MIRAMACS registry, which included 214 HeartMate 3 recipients, 5-year freedom from ischemic and hemorrhagic stroke was 91.7% and 88.6%, respectively, confirming that both event types remain clinically relevant during prolonged support [18]. A prespecified analysis of the MOMENTUM 3 trial further demonstrated that the incidence of late stroke beyond 6 months was lower with HeartMate 3 than with HeartMate II (0.04 vs. 0.13 events per patient-year; *p* = 0.01); nevertheless, stroke of either subtype and any functional severity was associated with substantially poorer 2-year survival [19]. The contribution of infection is further supported by a broader EUROMACS analysis of 3282 LVAD recipients, in which infection was independently associated with an approximately twofold increase in the risk of subsequent cerebrovascular events [20]. Ischemic stroke during mechanical circulatory support is therefore likely multifactorial and may be related to patient-specific thromboembolic risk, atrial arrhythmias, infection, hypertension, anticoagulation variability, procedural factors, or thrombus formation outside the pump. Hemorrhagic stroke similarly reflects the narrow therapeutic balance between preventing thromboembolism and avoiding excessive anticoagulation. Accordingly, long-term neurological risk reduction requires not only a hemocompatible pump but also meticulous blood pressure control, individualized antithrombotic management, aggressive infection prevention and treatment, and close multidisciplinary surveillance.

Bleeding complications also remained clinically relevant during extended support. Although the HeartMate 3 was designed to reduce shear stress and blood trauma, continuous-flow physiology, gastrointestinal angiodysplasia, antithrombotic therapy, and persistent abnormalities in von Willebrand factor collectively contribute to the residual bleeding risk. In the present cohort, crude patient-level occurrence proportions of gastrointestinal bleeding differed across age groups, occurring in 5.9%, 7.3%, and 23.8% of patients aged < 50, 50–64, and ≥65 years, respectively (global *p* = 0.025). However, these comparisons were not adjusted for exposure time or differences in duration of LVAD support and should therefore be interpreted as comparisons of patient-level occurrence proportions rather than differences in gastrointestinal bleeding incidence or risk. The higher crude proportion observed in the oldest subgroup should also be interpreted cautiously given the small number of patients aged ≥ 65 years and the absence of post-hoc pairwise comparisons. Klaeske et al. identified acquired von Willebrand syndrome in 97.1% of HeartMate 3 recipients, although the loss of high-molecular-weight von Willebrand factor multimers was less pronounced than that observed with the HeartMate II, indicating that improved pump hemocompatibility mitigates but does not eliminate this mechanism [21]. The occurrence of hemorrhagic stroke in six patients in our cohort further highlights the difficulty of maintaining an optimal antithrombotic balance over several years of support. Importantly, the international, randomized, double-blind ARIES-HM3 trial demonstrated that aspirin avoidance in patients receiving vitamin K antagonist therapy reduced nonsurgical bleeding events by 34% compared with aspirin-containing therapy, without increasing pump thrombosis, stroke, or other thromboembolic events [22]. These findings provide robust evidence that aspirin may be safely omitted from the routine antithrombotic regimen in appropriately selected HeartMate 3 recipients. Further reduction in anticoagulation intensity remains less well established. In the prospective MAGENTUM-1 pilot study, an INR target of 1.5–1.9 was evaluated in 15 closely monitored HeartMate 3 recipients; 14 patients remained free from pump thrombosis, disabling stroke, or major bleeding at 6 months, and no thrombotic events were observed [23]. However, the small sample size, single-arm design, intensive monitoring, and limited follow-up preclude broad adoption of this strategy. Therefore, while contemporary antithrombotic management is increasingly shifting toward bleeding reduction, particularly through aspirin avoidance, further modification of anticoagulation intensity should remain individualized and should account for each patient’s neurological, infectious, arrhythmic, vascular, and thrombotic risk profile.

Driveline infection remains one of the major unresolved concerns during long-term LVAD support. In the present cohort, driveline infection occurred in 45 patients (20.9%), with no significant difference across age groups. Although the HeartMate 3 has substantially reduced hemocompatibility-related adverse events, its percutaneous driveline continues to provide a potential route for microbial entry. A comprehensive analysis of the MOMENTUM 3 trial demonstrated that infections remained frequent and that infection-related outcomes were not substantially altered by pump type [24]. Similarly, the 2024 ISHLT consensus document continues to identify infection as a significant concern in patients receiving durable mechanical circulatory support [8]. More recently, the ASPIRE study, which included 3725 HeartMate 3 recipients from 30 centers, demonstrated considerable variation in driveline-care practices. The combined use of a chlorhexidine cleanser and silver-impregnated dressing was associated with lower risks of both early and late device-related infection, whereas frequent dressing changes were associated with a higher risk of late infection [25]. These findings emphasize the importance of standardized exit-site care, secure driveline anchoring, patient education, early recognition, and multidisciplinary treatment. Once driveline infection becomes deep, persistent, or recurrent, antimicrobial therapy and local wound care alone may be insufficient, and surgical source control may be required. Depending on the anatomical extent of infection and the condition of the surrounding soft tissues, treatment options may include surgical debridement, driveline repositioning or relocation, negative-pressure wound therapy, and coverage with well-vascularized tissue. Small surgical series have demonstrated that such individualized strategies may permit device salvage; however, persistent or recurrent infection may still occur despite apparently successful local treatment [26]. Device exchange or transplantation may therefore be considered in selected patients with refractory infection when durable source control cannot otherwise be achieved. Ultimately, further reduction in this complication may require fully implantable systems that eliminate the percutaneous driveline.

The increasing duration of support has also changed the clinical role of durable LVAD therapy. HeartMate 3 is no longer used solely as a temporary bridge to transplantation but increasingly functions as a long-term therapeutic platform for patients awaiting a donor organ and for those receiving destination therapy. In our cohort, some patients underwent successful heart transplantation after prolonged support, whereas others remained alive on device support for many years. This experience emphasizes that long-term outcomes should be evaluated not only through survival but also through freedom from disabling neurological events, recurrent bleeding, infection, device failure, repeated hospitalization, and reintervention. The evolving complication profile suggests that further improvements in LVAD outcomes will increasingly depend on long-term medical management, driveline infection prevention, surveillance of the outflow graft, and the future development of fully implantable systems.

Right heart failure was the most frequent major nonfatal adverse event in our cohort, occurring in 55 patients (25.5%). Its frequency increased across the predefined age groups, from 20.0% in patients aged < 50 years to 25.7% in those aged 50–64 years and 47.6% in those aged ≥ 65 years (*p* = 0.036). However, this finding should be interpreted cautiously because the age-stratified analysis was exploratory and the oldest group included only 21 patients. In a contemporary analysis of 2200 HeartMate 3 recipients from the MOMENTUM 3 portfolio, early right ventricular failure occurred in 18.3%, including 6.4% who required temporary right ventricular assist device support, and was independently associated with substantially increased mortality [27]. Right heart failure after LVAD implantation is multifactorial and may reflect limited preoperative right ventricular reserve compounded by increased venous return, interventricular septal shift, perioperative myocardial injury, arrhythmias, or persistent pulmonary vascular load. These findings reinforce the importance of comprehensive preoperative assessment of right ventricular function and hemodynamics, optimization of volume status and pulmonary pressures, avoidance of excessive left ventricular unloading, and early escalation to pharmacological or temporary mechanical support when right ventricular deterioration occurs [1]. Importantly, durable LVAD support unloads the left ventricle but does not directly support the right ventricle. Persistent or progressive right ventricular dysfunction may therefore impair left ventricular filling, reduce effective LVAD flow, and limit systemic perfusion despite otherwise adequate left ventricular unloading. In selected patients at high risk of perioperative right ventricular failure, inotropic or inodilator therapy, including levosimendan, has been investigated as an adjunctive strategy; however, available observational data are inconsistent, and a definitive benefit in preventing right ventricular failure or improving survival has not been established [28,29]. Long-term management consequently requires continued assessment of volume status, pulmonary vascular load, right ventricular function, and LVAD speed to maintain an appropriate balance between left ventricular unloading and right-sided filling.

Clinically significant arrhythmias during follow-up were observed in 23 patients (10.7%) in our cohort. Ventricular arrhythmias nevertheless represent an important challenge during durable LVAD support because mechanical left ventricular support does not necessarily ensure hemodynamic tolerance of sustained ventricular tachycardia. Particularly in patients with impaired right ventricular function, sustained or recurrent ventricular arrhythmias may reduce right ventricular output, diminish left ventricular preload and LVAD filling, and ultimately compromise systemic perfusion. Although ICD therapy may terminate malignant ventricular arrhythmias, recurrent shocks may impose substantial morbidity without correcting the underlying hemodynamic or arrhythmogenic substrate. Contemporary consensus recommendations therefore support an individualized approach incorporating correction of reversible triggers, assessment for suction or excessive ventricular unloading, optimization of LVAD speed and hemodynamics, individualized ICD programming, and antiarrhythmic therapy. Catheter ablation should be considered in selected patients with recurrent or drug-refractory ventricular arrhythmias, particularly when repeated ICD therapies or hemodynamic deterioration occur [30,31].

As the duration of LVAD support increases, successful outcomes depend not only on device performance but also on the patient’s ability to adapt to device-supported life and maintain complex self-care behaviors. Dependence on an external controller and power supply, driveline care, anticoagulation, alarm management, regular follow-up, and emergency planning imposes substantial cognitive, emotional, and practical demands on both patients and caregivers. ISHLT recommendations therefore emphasize comprehensive psychosocial assessment, evaluation of health literacy and social support, and continued monitoring of psychological well-being and adherence before and after implantation [1,32]. In an INTERMACS analysis of 15,403 continuous-flow LVAD recipients, psychosocial risk factors including limited social support, cognitive impairment, severe psychiatric disease and repeated nonadherence were associated with higher risks of device-related infection, gastrointestinal bleeding, pump thrombosis, and hospital readmission, although on-device survival was not significantly different [33]. Accordingly, repeated competency-based education, active caregiver involvement, assessment and treatment of anxiety or depression, and ready access to LVAD coordinators, social workers, and mental health professionals should be regarded as integral components of long-term LVAD care rather than as adjunctive measures.

Remote monitoring may represent an additional strategy to support longitudinal care as the duration of LVAD support continues to increase. Contemporary evidence in heart failure suggests that remote monitoring incorporating home-based devices, implantable sensors, and digital applications may facilitate earlier recognition of clinical deterioration and enable timely intervention [34]. In patients receiving durable LVAD support, integration of remote monitoring into multidisciplinary follow-up may therefore be particularly valuable for the surveillance of clinical status and device-related complications. However, dedicated prospective evidence in LVAD recipients remains limited, and the optimal structure and clinical impact of such monitoring strategies require further evaluation.

Several limitations should be acknowledged. First, this was a retrospective, single-center observational study and was therefore subject to selection bias, incomplete documentation, and unmeasured confounding. Second, although the cohort represents a substantial institutional experience with prolonged follow-up, its sample size was smaller than those of multicenter registries and randomized trials, limiting the precision of subgroup analyses. Third, patient selection, surgical techniques, anticoagulation protocols, blood pressure targets, infection-prevention strategies, and postoperative management evolved during the decade-long study period. Fourth, female patients represented only 7.9% of the cohort, and no women were included in the ≥65-year subgroup, limiting the generalizability of the findings, particularly for age-stratified analyses. In addition, a substantial proportion of patients remained alive on ongoing HeartMate 3 support at the data cutoff, resulting in considerable censoring and reduced precision of estimates at later follow-up time points. The age-stratified analysis was particularly limited by the small number of patients aged ≥ 65 years, with only four patients remaining at risk at 5 years. Important baseline characteristics, including treatment strategy, ischemic etiology, renal function, and hemoglobin level, differed across age groups, limiting causal interpretation of age-related comparisons. The multivariable Cox model included eight predictor effects for 58 on-device deaths (crude events-per-predictor ratio, 7.25). Accordingly, the number of events remained limited relative to the eight estimated predictor effects, and coefficient instability and overfitting cannot be excluded. No adjustment for multiple comparisons was performed for the exploratory secondary and age-stratified analyses, increasing the possibility of chance findings. Although the proportional hazards assumption was not violated, the model demonstrated only modest discrimination (Harrell C-statistic, 0.648); therefore, the multivariable findings should be interpreted primarily as exploratory associations, and the model should not be regarded as a prognostic prediction tool. Finally, long-term adverse events were recorded primarily according to whether at least one event occurred during HeartMate 3 support. Exact timing and recurrent-event burden were not consistently available for all nonfatal outcomes; therefore, these data are presented as patient-level occurrence rather than incidence rates or formal time-to-event comparisons. Differences in support duration should consequently be considered when interpreting age-group comparisons of adverse events.

## 5. Conclusions

This nearly decade-long experience demonstrates that HeartMate 3 provides durable and reliable long-term mechanical circulatory support. Transplant-censored on-device survival remained favorable. Higher preoperative creatinine was independently associated with on-device mortality. No statistically significant association between age and on-device mortality was detected in the present analysis; however, the small number of older recipients and limited long-term risk set preclude conclusions regarding the absence of an age-related effect. These findings further support the role of HeartMate 3 as a long-term therapeutic option for appropriately selected patients with advanced heart failure.

## Figures and Tables

**Table 1:** Baseline characteristics according to age group.

Variable	Overall Cohort (n = 215)	<50 Years (n = 85)	50–64 Years (n = 109)	≥65 Years (n = 21)	*p* Value
Age, years	53 (45–60)	42 (36–47)	57 (54–60)	67 (66–69)	**<0.001**
Female sex	17 (7.9)	7 (8.2)	10 (9.2)	0 (0.0)	0.358
Height, cm	174 (169–178)	174 (168–180)	173 (169–176)	175 (169–178)	0.611
Weight, kg	78 (70–86)	80 (70–88)	77 (70–86)	78 (70–88)	0.946
BMI, kg/m^2^	25.7 (23.7–28.4)	25.9 (23.5–28.6)	25.6 (23.9–28.7)	25.2 (24.0–27.8)	0.812
BSA, m^2^	1.93 (1.82–2.06)	1.97 (1.82–2.08)	1.91 (1.82–2.04)	1.91 (1.81–2.05)	0.817
**Etiology**					**0.003**
Dilated cardiomyopathy	135 (62.8)	67 (78.8)	59 (54.1)	9 (42.9)	
Ischemic cardiomyopathy	77 (35.8)	17 (20.0)	48 (44.0)	12 (57.1)	
Arrhythmogenic right ventricular dysplasia	1 (0.5)	1 (1.2)	0 (0.0)	0 (0.0)	
Valvular cardiomyopathy	2 (0.9)	0 (0.0)	2 (1.8)	0 (0.0)	
**INTERMACS profile**					0.126
INTERMACS profile 1	13 (6.0)	7 (8.2)	3 (2.7)	3 (14.3)	
INTERMACS profile 2	49 (22.8)	23 (27.1)	22 (20.2)	4 (19.0)	
INTERMACS profile 3	75 (34.9)	27 (31.8)	43 (39.4)	5 (23.8)	
INTERMACS profile 4	78 (36.3)	28 (32.9)	41 (37.6)	9 (42.9)	
**LVAD strategy at implantation**					**<0.001**
Bridge to transplantation	160 (74.4)	80 (94.1)	78 (71.6)	2 (9.5)	
Bridge to decision	35 (16.3)	5 (5.9)	23 (21.1)	7 (33.3)	
Destination therapy	20 (9.3)	0 (0.0)	8 (7.3)	12 (57.1)	
LVEF, %	20 (15–20)	19 (17–20)	20 (15–20)	19 (15–20)	0.533
sPAP, mmHg	45 (40–55)	42 (36–50)	48 (40–60)	46 (40–58)	**0.043**
LVESD, cm	6.3 (5.7–6.8)	6.5 (5.8–6.8)	6.1 (5.5–6.6)	6.1 (5.8–7.0)	0.061
LVEDD, cm	6.9 (6.3–7.5)	7.1 (6.6–7.7)	6.7 (6.3–7.3)	6.9 (6.4–7.4)	0.069
RV diameter, cm	2.9 (2.6–3.2)	3.0 (2.6–3.4)	2.9 (2.5–3.2)	3.0 (2.4–3.2)	0.428
Creatinine, mg/dL	1.05 (0.89–1.33)	0.93 (0.86–1.20)	1.10 (0.90–1.37)	1.40 (1.05–1.60)	**0.001**
eGFR, mL/min/1.73 m^2^	80.0 (58.1–85.0)	80.0 (75.0–90.0)	75.0 (56.0–85.0)	55.0 (42.9–80.0)	**<0.001**
Hemoglobin, g/dL	12.2 (10.8–13.8)	13.2 (11.6–14.2)	11.9 (10.4–13.4)	11.4 (10.2–13.0)	**0.003**
Preoperative inotropic support	51 (23.7)	27 (31.8)	21 (19.3)	3 (14.3)	0.072
ICD	129 (60.0)	47 (55.3)	70 (64.2)	12 (57.1)	0.435
CRT	6 (2.8)	4 (4.7)	1 (0.9)	1 (4.8)	0.239

Data are presented as median (IQR) or n (%). *p* values represent global comparisons across the three age groups using the Kruskal–Wallis test or Pearson chi-square/Fisher–Freeman–Halton exact test, as appropriate. BMI, body mass index; BSA, body surface area; CRT, cardiac resynchronization therapy; ICD, implantable cardioverter–defibrillator; INTERMACS, Interagency Registry for Mechanically Assisted Circulatory Support; LVEDD, left ventricular end-diastolic diameter; LVEF, left ventricular ejection fraction; LVESD, left ventricular end-systolic diameter; RV, right ventricular; sPAP, systolic pulmonary artery pressure. Bold text indicates category/subgroup headings; bold *p* values indicate statistical significance (*p* < 0.05).

**Table 2:** Follow-up status and device-related adverse events according to age group.

Variable	Overall Cohort (n = 215)	<50 Years (n = 85)	50–64 Years (n = 109)	≥65 Years (n = 21)	*p* Value
**Follow-up status**					0.216
Alive on ongoing LVAD support	149 (69.3)	62 (72.9)	71 (65.1)	16 (76.2)	
Heart transplantation	8 (3.7)	5 (5.9)	2 (1.8)	1 (4.8)	
Death on LVAD support	58 (27.0)	18 (21.2)	36 (33.0)	4 (19.0)	
Device failure	7 (3.3)	5 (5.9)	2 (1.8)	0 (0.0)	0.195
Gastrointestinal bleeding	18 (8.4)	5 (5.9)	8 (7.3)	5 (23.8)	**0.025**
Ischemic stroke	8 (3.7)	3 (3.5)	5 (4.6)	0 (0.0)	1.000
Hemorrhagic stroke	6 (2.8)	0 (0.0)	5 (4.6)	1 (4.8)	0.133
Major neurological event	14 (6.5)	3 (3.5)	10 (9.2)	1 (4.8)	0.270
Driveline infection	45 (20.9)	19 (22.4)	24 (22.0)	2 (9.5)	0.400
Arrhythmia during follow-up	23 (10.7)	14 (16.5)	8 (7.3)	1 (4.8)	0.081

Data are presented as median (IQR) or n (%). *p* values represent global comparisons across the three age groups using the Kruskal–Wallis test or Pearson chi-square/Fisher–Freeman–Halton exact test, as appropriate. Device failure includes outflow graft obstruction and other mechanical complications and does not represent intrinsic pump thrombosis. Adverse events are reported as the number and percentage of patients who experienced at least one event during device support. Bold text indicates category/subgroup headings; bold *p* values indicate statistical significance (*p* < 0.05).

**Table 3:** Multivariable Cox regression analysis of transplant-censored on-device mortality.

Variable	Hazard Ratio	95% CI	*p* Value
Age, per 10-year increase	1.15	0.87–1.52	0.338
Female sex	0.58	0.14–2.51	0.468
Ischemic cardiomyopathy	0.88	0.48–1.62	0.679
INTERMACS profile 1–2	0.76	0.42–1.39	0.379
Preoperative creatinine, per 1 mg/dL increase	1.93	1.07–3.50	**0.029**
Preoperative hemoglobin, per 1 g/dL increase	0.88	0.76–1.01	0.076
Preoperative inotropic support	1.52	0.85–2.72	0.162
Implantation year, per 1-year increase	0.93	0.84–1.02	0.122

For the cause-specific Cox analysis, patients were censored at heart transplantation. All listed covariates were included simultaneously. CI, confidence interval; INTERMACS, Interagency Registry for Mechanically Assisted Circulatory Support. Harrell C-statistic for model discrimination: 0.648. bold *p* values indicate statistical significance (*p* < 0.05).

## Data Availability

The data that support the findings of this study are available from the corresponding author upon reasonable request. The data are not publicly available due to ethical and privacy restrictions related to patient confidentiality.

## Supplementary Materials

The following supporting information can be downloaded at https://www.mdpi.com/article/10.3390/life16101640/s1, Table S1: Operative and early postoperative outcomes according to age group; Figure S1: STROBE study flow diagram; Supplementary File S1. Completed STROBE Statement—Checklist of items that should be included in reports of cohort studies.
