# Supplementary material for: A Decade of Durable Mechanical Circulatory Support with HeartMate 3 Across Age Groups in a Single-Center Experience
Source: Life (Basel). 2026 Sep 30;16(10):1640. doi: 10.3390/life16101640 (PMC13705987; doi:10.3390/life16101640)
Supplement: Supplementary file 1 [file life-16-01640-s001.zip › Supplementary Figure S1.pdf]

Study Flow Diagram

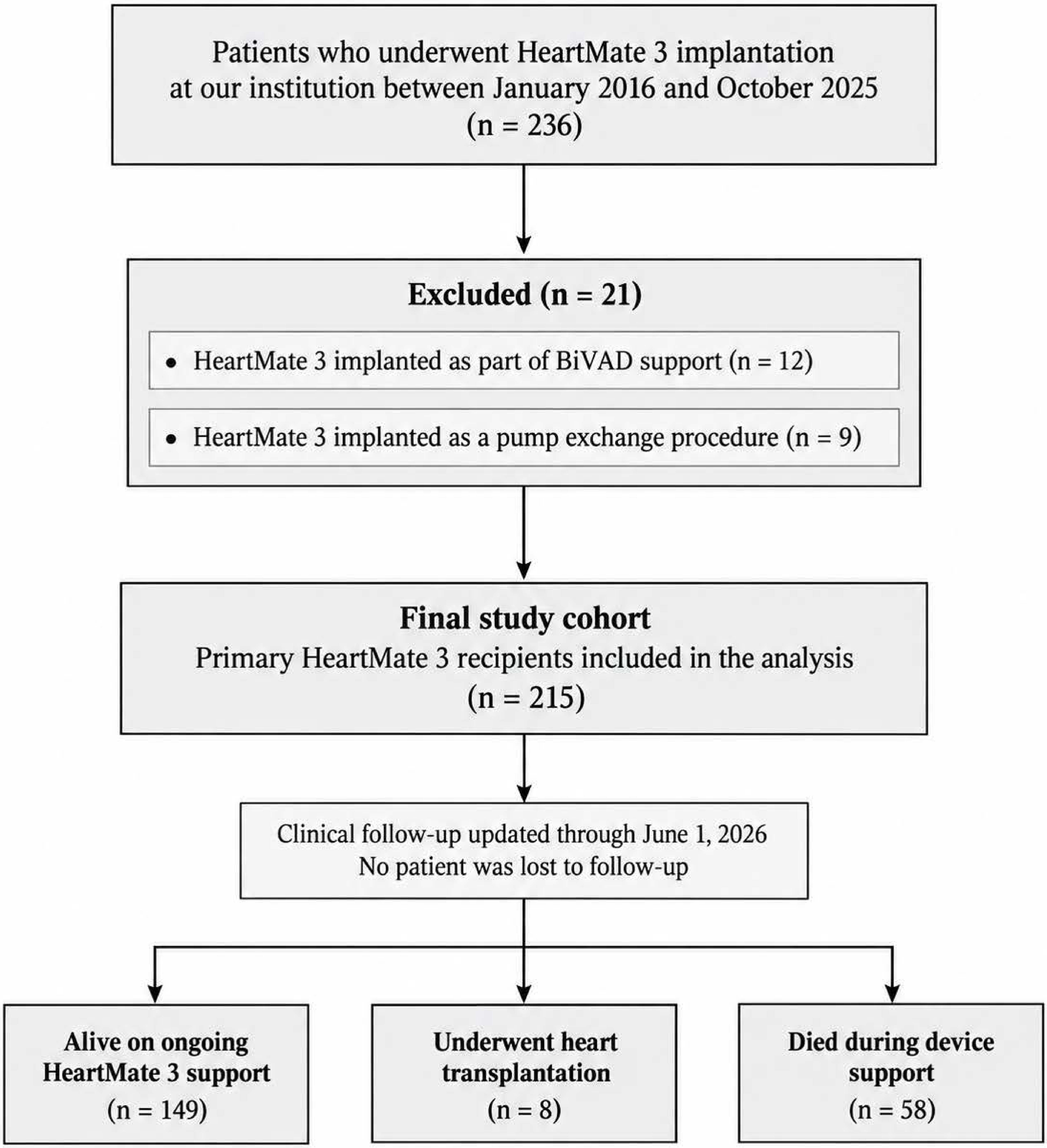

BiVAD, biventricular assist device.
