# Supplementary material for: A Decade of Durable Mechanical Circulatory Support with HeartMate 3 Across Age Groups in a Single-Center Experience
Source: Life (Basel). 2026 Sep 30;16(10):1640. doi: 10.3390/life16101640 (PMC13705987; doi:10.3390/life16101640)
Supplement: Supplementary file 1 [file life-16-01640-s001.zip › Supplementary File S1-STROBE Checklist HM3.pdf]

# STROBE Statement - Checklist of Items for Cohort Studies

## A Decade of Durable Mechanical Circulatory Support with HeartMate 3 Across Age Groups in a Single-Center Experience

| Section | Item | STROBE recommendation | Where reported in manuscript |
|---|---|---|---|
| Title and abstract | 1a | Identify the study design using a commonly used term in the title or abstract. | Abstract (Methods): retrospective single-center cohort study |
| Title and abstract | 1b | Provide an informative and balanced summary of the methods and main findings. | Abstract |
| Introduction - Background/rationale | 2 | Explain the scientific background and rationale for the investigation. | Introduction, background/rationale paragraphs |
| Introduction - Objectives | 3 | State the study objectives and any prespecified hypotheses. | Introduction, final paragraph; study objective and prespecified hypothesis |
| Methods - Study design | 4 | Present the key elements of the study design early in the manuscript. | Section 2.1, Study Design and Patient Population |
| Methods - Setting | 5 | Describe the setting and relevant dates, including recruitment and follow-up periods. | Section 2.1; high-volume tertiary heart failure/MCS center; implantation January 2016-October 2025; follow-up through June 1, 2026 |
| Methods - Participants | 6a | Describe eligibility criteria, participant selection, and follow-up methods. | Sections 2.1-2.2; 236 HeartMate 3 implants screened; 12 BiVAD and 9 pump-exchange cases excluded; final cohort n=215; structured LVAD-clinic follow-up |
| Methods - Participants | 6b | For matched cohort studies, report matching criteria and numbers in each group. | Not applicable; this was not a matched cohort study |
| Methods - Variables | 7 | Define outcomes, predictors, potential confounders, effect modifiers, and diagnostic criteria where applicable. | Sections 2.5-2.6; primary/secondary outcomes and adverse-event definitions; Section 2.7, prespecified Cox covariates |
| Methods - Data sources/measurement | 8 | Describe data sources and methods of assessment for variables of interest. | Section 2.2; electronic medical records, institutional MCS database, scheduled LVAD-clinic visits, and institutional hospital records |
| Methods - Bias | 9 | Describe efforts to address potential sources of bias. | Sections 2.1, 2.2, and 2.7; consecutive eligible cohort, complete final device status/no loss to follow-up, prespecified covariates, competing-risk framework for transplantation, and exploratory age-stratified analyses |
| Methods - Study size | 10 | Explain how the study size was determined. | Section 2.7; all consecutive eligible patients during the prespecified study period were included; no formal a priori sample-size calculation |
| Methods - Quantitative variables | 11 | Explain handling of quantitative variables and justify categorization where applicable. | Section 2.7; continuous variables summarized as median (IQR); age categorized as <50, 50-64, and >=65 years for prespecified exploratory analyses and also modeled continuously per 10-year increase; rationale for cut-points stated in Methods |
| Methods - Statistical methods | 12a | Describe statistical methods, including approaches used to control for confounding. | Section 2.7; Kruskal-Wallis, Pearson chi-square/Fisher-Freeman-Halton, Aalen-Johansen cumulative incidence, Fine-Gray regression, Kaplan-Meier, and multivariable cause-specific Cox regression; prespecified covariates entered simultaneously |
| Methods - Statistical methods | 12b | Describe methods used for subgroup or interaction analyses. | Section 2.7; prespecified exploratory age-stratified analyses; no formal interaction analysis was performed |
| Methods - Statistical methods | 12c | Explain how missing data were addressed. | Section 2.7 and Section 3.6; no imputation, analyses based on available data; the final Cox model included all 215 patients, indicating no missing data for included covariates |
| Methods - Statistical methods | 12d | Explain how loss to follow-up was addressed, if applicable. | Section 2.2; no patient was lost to follow-up and final device status was available for all 215 patients |
| Methods - Statistical methods | 12e | Describe sensitivity analyses, if performed. | Section 2.7; no formal sensitivity analysis was prespecified. A secondary transplant-censored Kaplan-Meier analysis was performed as a complementary descriptive analysis to the primary competing-risk framework |
| Results - Participants | 13a | Report numbers of participants at each stage of the study. | Section 3.1 and Supplementary Figure S1; 236 screened, 21 excluded, 215 analyzed |
| Results - Participants | 13b | Give reasons for exclusions/non-participation at each stage. | Section 2.1 and Supplementary Figure S1; BiVAD support (n=12) and pump exchange (n=9) |
| Results - Participants | 13c | Consider presenting a participant flow diagram. | Supplementary Figure S1 |
| Results - Descriptive data | 14a | Report participant characteristics and relevant exposures/confounders. | Table 1 |
| Results - Descriptive data | 14b | Indicate the number of participants with missing data for variables of interest. | Section 2.7 and Section 3.6; no imputation; final multivariable model included all 215 participants with no missing data for included covariates |
| Results - Descriptive data | 14c | Summarize follow-up time. | Section 3.1; observed support duration and reverse |

| | | | |
|---|---|---|---|
| | | | Kaplan-Meier follow-up reported (median support 603 days; median potential follow-up 1061 days) |
| Results - Outcome data | 15 | Report numbers of outcome events or summary measures over time. | Section 3.3 and Supplementary Table S1; Section 3.4 and Table 2; Section 3.5/Figures 1-2; postoperative outcomes, deaths/transplantation, major adverse events, cumulative incidence, and time-specific survival estimates reported |
| Results - Main results | 16a | Provide unadjusted and adjusted estimates with precision and identify adjusted covariates. | Section 3.6 and Table 3; adjusted HRs with 95% CIs reported; covariates identified in Section 2.7. Unadjusted/descriptive age-group comparisons are reported in Tables 1-2 and Supplementary Table S1 |
| Results - Main results | 16b | Report category boundaries when continuous variables are categorized. | Section 2.7, Table 1, Supplementary Table S1, and Table 2; age categories <50, 50-64, and >=65 years are explicitly reported |
| Results - Main results | 16c | When relevant, consider translating relative estimates into absolute risk over a meaningful period. | Section 3.5 and Figures 1-2; 5-year cumulative incidences of on-device death/transplantation and 30-day, 1-year, and 5-year survival estimates reported |
| Results - Other analyses | 17 | Report other analyses such as subgroup, interaction, or sensitivity analyses. | Sections 3.5-3.6; exploratory age-stratified Fine-Gray analysis, secondary transplant-censored Kaplan-Meier analysis, and assessment of continuous-covariate functional form/proportional hazards |
| Discussion - Key results | 18 | Summarize key results in relation to the study objectives. | Discussion, opening paragraph |
| Discussion - Limitations | 19 | Discuss limitations, including potential bias and imprecision. | Discussion, Limitations paragraph; retrospective single-center design, small >=65-year subgroup, substantial late censoring, baseline imbalances, modest event-to-predictor ratio/model discrimination, no multiplicity adjustment for exploratory analyses, and non-time-normalized adverse-event ascertainment |
| Discussion - Interpretation | 20 | Provide a cautious interpretation considering objectives, limitations, multiplicity, and other evidence. | Discussion; interpretation is explicitly cautious regarding age effects, late follow-up precision, competing treatment pathways, multiplicity, and comparison with randomized/registry evidence |
| Discussion - Generalisability | 21 | Discuss the generalisability/external validity of the findings. | Discussion, age-stratified interpretation and Limitations/Conclusion; single-center case mix, low female representation, small oldest subgroup, and external-validity constraints are addressed |
| Other information - Funding | 22 | State the source of funding and the role of funders. | Funding statement: no external funding |
