# Supplementary material for: A Decade of Durable Mechanical Circulatory Support with HeartMate 3 Across Age Groups in a Single-Center Experience
Source: Life (Basel). 2026 Sep 30;16(10):1640. doi: 10.3390/life16101640 (PMC13705987; doi:10.3390/life16101640)
Supplement: Supplementary file 1 [file life-16-01640-s001.zip › Supplementary Table S1.pdf]

Supplementary Table S1. *Operative and early postoperative outcomes according to age group*

**Variable**	**Overall cohort (n=215)**	**<50 years (n=85)**	**50-64 years (n=109)**	**>=65 years (n=21)**	**P value**
Cardiopulmonary bypass time, min	69 (57-84)	69 (57-85)	67 (56-81)	72 (63-101)	0.237
Redo sternotomy	28 (13.0)	7 (8.2)	18 (16.5)	3 (14.3)	0.232
Intraoperative RBC transfusion, units	0 (0-1)	0 (0-1)	0 (0-1)	0 (0-1)	0.501
Intraoperative nitric oxide support	106 (49.3)	39 (45.9)	52 (47.7)	15 (71.4)	0.105
Intraoperative IABP support	14 (6.5)	8 (9.4)	4 (3.7)	2 (9.5)	0.231
Intraoperative VA-ECMO support	6 (2.8)	1 (1.2)	5 (4.6)	0 (0.0)	0.257
CentriMag® support for RHF	4 (1.9)	2 (2.4)	2 (1.8)	0 (0.0)	0.774
Postoperative hemorrhage >1000 mL	24 (11.2)	5 (5.9)	15 (13.8)	4 (19.0)	0.108
Reoperation/revision	29 (13.5)	12 (14.1)	15 (13.8)	2 (9.5)	0.853
Mechanical ventilation duration, h	17 (15-20)	17 (15-20)	17 (15-20)	19 (15-27)	0.299
Prolonged ventilation >48 h	24 (11.2)	7 (8.2)	14 (12.8)	3 (14.3)	0.535
Peak postoperative lactate, mmol/L	6.4 (4.6-8.7)	6.2 (4.6-8.1)	6.3 (4.6-9.5)	7.2 (6.0-8.5)	0.509
Non-dialysis AKI	11 (5.1)	4 (4.7)	5 (4.6)	2 (9.5)	0.478
Dialysis-requiring AKI	20 (9.3)	6 (7.1)	11 (10.1)	3 (14.3)	0.504
Low cardiac output syndrome	28 (13.0)	11 (12.9)	13 (11.9)	4 (19.0)	0.635
Early postoperative right heart failure	55 (25.5)	17 (20.0)	28 (25.7)	10 (47.6)	**0.036**
Postoperative RBC transfusion, units	1 (0-2)	1 (0-2)	1 (1-3)	1 (0-3)	0.229
Postoperative FFP transfusion, units	2 (1-4)	2 (1-3)	2 (1-4)	2 (1-4)	0.782
Postoperative platelet transfusion, units	0 (0-0)	0 (0-0)	0 (0-0)	0 (0-0)	0.977
Early Postoperative arrhythmia	48 (22.3)	15 (17.6)	27 (24.8)	6 (28.6)	0.383
Postoperative IABP support	13 (6.0)	7 (8.2)	4 (3.7)	2 (9.5)	0.325
Postoperative nitric oxide support	106 (49.3)	38 (44.7)	53 (48.6)	15 (71.4)	0.090
Postoperative ECMO support	8 (3.7)	3 (3.5)	5 (4.6)	0 (0.0)	0.592
ICU stay, days	9 (7-13)	10 (7-13)	9 (7-11)	10 (7-17)	0.877
Hospital stay, days	23 (19-30)	23 (19-28)	23 (18-30)	24 (20-27)	0.951
30-day mortality	27 (12.6)	7 (8.2)	17 (15.6)	3 (14.3)	0.298

Data are presented as median (IQR) or n (%). P values represent global comparisons across the three age groups using the Kruskal-Wallis or chi-square test, as appropriate. AKI, acute kidney injury; ECMO, extracorporeal membrane oxygenation; FFP, fresh frozen plasma; IABP, intra-aortic balloon pump; ICU, intensive care unit; RBC, red blood cell; VA-ECMO, venoarterial extracorporeal membrane oxygenation; RHF, right heart failure, CentriMag® Ventricular Assist System, Thoratec Corp., Pleasanton, CA, USA..
